# Remarks on a Scaling Theory of Spread of COVID-19 with an Application to the Case of Bulgaria

**DOI:** 10.3390/e28010082

**Published:** 2026-01-10

**Authors:** Svetlan Kartalov, Nikolay K. Vitanov

**Affiliations:** Institute of Mechanics, Bulgarian Academy of Sciences, Acad. Georgi Bonchev Str., Block 4, 1113 Sofia, Bulgaria; kartalov@abv.bg

**Keywords:** self-similarity, allometric urban scaling, dynamic scaling, power law, SARS-CoV-2, COVID-19, homocrony number, Bulgaria

## Abstract

We present several remarks on the spread of the COVID-19 epidemics in Bulgaria. The remarks are based on the hypothesis that the spread of the infection exhibits scaling properties similar to the scaling in urban dynamics. The corresponding mathematical theory leads us to a relationship for a power-law dependence of the number of infected in a certain region on the corresponding homochrony number. We prove the correctness of the mathematical theory on the basis of data for several Bulgarian regions for the first large COVID-19 wave in 2020. We observe a collapse of the real data along a single straight line.

## 1. Introduction

Complex systems and their nonlinearity have attracted much research attention in recent decades [1,2,3,4,5,6,7,8,9,10,11,12]. Especially interesting are the various kinds of network models which can be connected to migration, the spread of epidemics and to other kinds of flows in networks [13,14,15,16,17,18,19,20]. The spread of epidemics has been a much-studied area of science for many years [21,22,23,24,25,26,27,28,29,30,31,32,33,34,35,36,37]. There exist various kinds of mathematical models which model different aspects of epidemic spread. Many of these models are deterministic [29,38,39,40,41]. Some of these models use a methodology for obtaining exact solutions of nonlinear differential equations [42,43,44,45,46,47,48]. Other models are stochastic [49,50,51,52,53]. The third class of models is based on Monte Carlo simulations [54,55,56]. In this text we discuss a model based on the concept of scaling. Examples for this class of models can be found in [57,58,59,60,61,62,63,64].

Below, we present several remarks about the COVID-19 spread process. We note the following about this study.

1. We assume that the process has a specific property: a self-similarity is presented in the course of the spread of the infection.
2. The above assumption allows us to construct an efficient model with a minimum number of parameters and a low level of mathematical complexity.
3. The model is effective for growth dynamics of the number of established cases that are different in their characteristics, but similar in their structure of cohorts and regions.

Scale-invarince, self-similarity and scaling are closely connected to the power laws we encounter in physics and other areas of natural sciences [58,65,66,67,68,69,70,71,72,73,74,75,76,77,78,79,80,81,82,83,84,85,86,87,88]. On the basis of this property, one can use Buchingam’s Pi-theorem in order to study the dependence of dimensionless governed parameters on other dimensionless governing parameters. Several remarks on the self-similarity and scaling follow.

1. Two phenomena are similar if they differ only in the numerical values of their dimensional governing parameters when the values of their dimensionless governing parameters are identical.
2. If the two phenomena are one and the same phenomenon, then we can speak about self-similarity. Self-similarity can alo exist with respect to time, and this leads us to the concept of dynamic scaling.
3. A function f(x,t) exhibits dynamic scaling (Family-Vicsek scaling) if
  (1) $$f\left(x,t\right)\approx t^{\theta }\phi \left(\frac{x}{t^{z}}\right),$$
  where θ and *z* are parameters. We will use the concept of dynamic scaling below in the text.
4. Power laws are connected to scale invariance. A function f(r) is called scale-invariant or scale-free if it satisfies
  (2) f(λr)=g(λ)f(r),
  for any value of λ. In (2), g(λ) is a function. It can be proven that f(r) which satisfies (2) has the power-law form $f\left(r\right)∼r^{-\alpha }$, where α is a parameter. The form of g(λ) is $g\left(\lambda \right)∼\lambda^{p}$, where *p* is a parameter which is equal to $g^{\prime }\left(1\right)$.
5. A phenomenon is called an automodel phenomenon if its spatial characteristics change in time in such a way that the phenomenon remains similar geometrically:
  (3) $$U\left(x,t\right)=U_{0}\left(t\right)\;\;F\left[\frac{x}{x_{0}\left(t\right)}\right].$$
  In other words, there exist time-dependent scales, $U_{0}\left(t\right)$ and $x_{0}\left(t\right)$, such that the phenomenon is stationary as measured by these scales. The scaling windows for the quantity *x* and the time *t* are the intervals $x_{1}<<x<<x_{2}$ and $t_{1}<<t<<t_{2}$ in which (3) holds.

The new points in the research in this text are as follows. We systematically introduce the Family–Vicsek scaling approach for analysis of the characteristics of the front of a COVID-19 wave. We formulate a scaling hypothesis for COVID-19 spread and show that in the area of the scaling window, the number of infected follows a power law with respect to the homocrony number Ho. We validate this hypothesis on the basis of the Buckingham Pi-theorem. We check the hypothesis using the real data from the first large COVID-19 wave in Bulgaria and observe a clear collapse of the data from eight large areas to a single line that represents a power law of the homocrony number with scaling exponent $Ho^{1/3}$.

The text below is organized as follows. In Section 2, we present a scaling hypothesis for COVID-19 spread and a consequence of this hypothesis connected to a power law dependence on a quantity called the homocrony number. In Section 3, we show the correctness of the hypothesis on the basis of data for the spread of COVID-19 in Bulgaria. Several concluding remarks are summarized in Section 4.

## 2. The Scaling Hypothesis for COVID-19 Spread

The hypothesis about the dynamics of the spread of COVID-19 is that the spread dynamics obey the scaling laws known from the theory of urban scaling [57,89,90,91,92,93].

These scaling laws are expressed as power functions of two main parameters:

1. The population size.
2. The time since the beginning of the epidemic in the local community.

Within the scope of the theory of urban scaling, any numerical characteristic of an urban system can be represented in the form

(4) $$Y\left(N,t\right)=Y_{0}\left(t\right)N{\left(t\right)}^{\beta }\exp\left[\xi \left(t\right)\right].$$

The quantities in (4) represent the following characteristics of the urban system.

1. N(t): The population of the urban system.
2. $Y_{0}\left(t\right)$: A basic factor which is common for all elements of the urban system.
3. β: A dimensionless scaling exponent.
4. ξ(t): The deviation of the characteristic from the power law.

Then, for fixed *t*

(5) $$\ln Y=\ln Y_{0}+\beta \ln N+\xi .$$

The scaling exponent β can be considered as a measure of the density of the social networks of the individuals in the urban system.

Next, we formulate a hypothesis about the spread of COVID-19 in an urban system.

**COVID-19 Spread Hypothesis**:  *The dynamics of the spread of COVID-19 in an urban system with a population N are given by*

(6) f(C,N,t,τ)=0,

*where*

*1.*  *C: The total number of registered cases.*
*2.*  *N: The population of the urban system.*
*3.*  *t: The time of spread of COVID-19.*
*4.*  *τ: The characteristic time of the spread of COVID-19 in an urban system of population N.*

*The form of the function f is such that $C\propto NHo^{\beta }$, where Ho is the number of homochrony Ho=t/τ=(δt)/N and δ is an appropriate coefficient.*

Next, we will obtain several relationships on the basis of the above hypothesis. We introduce the following units for measurements:

1. Unit for measurement of humans: *H* (for measurement of population *N* and for registered cases *C*).
2. Unit for time: *T*.

We will proceed as follows. First, we apply the Buckingham Pi-theorem in order to obtain a form for the relationship between the number of infected *C* and the total population *N*. Then, we introduce the homocrony number Ho and show that the number of infected individuals is proportional to a power function of this number.

### 2.1. The Application of the Buckingham Pi-Theorem

We apply the Buckingham Pi-theorem. According to this theorem, the number of dimensionless groups which can be built is π=n−j. *n* is the number of quantities. *j* is the number of independent quantities. In our case, the number of quantities is four: C,N,t and τ. The number of independent quantities is two: *N* and *t*. According to the Buckingham Pi-theorem, n=4, j=2 and the number of dimensionless groups which can be built is π=n−j=2. These dimensionless groups are

(7) $$\begin{array}{c} \pi_{1}=N^{a_{1}}t^{b_{1}}C,\;\;\;\;\pi_{2}=N^{a_{2}}t^{b_{2}}\tau . \end{array}$$

$a_{1},a_{2},b_{1},b_{2}$ are parameters. On the basis of (7) we can write the equations for the dimensions:

(8) $$\begin{array}{c} H^{0}T^{0}=H^{a_{1}}T^{b_{1}}H,\;\;\;\;H^{0}T^{0}=H^{a_{2}}T^{b_{2}}T. \end{array}$$

From these equations, we obtain $a_{1}=-1$, $b_{1}=0$, $a_{2}=0$, $b_{2}=-1$. Then,

(9) $$\begin{array}{c} \pi_{1}=C/N,\;\;\;\;\pi_{2}=\tau /t. \end{array}$$

The function of the dimensionless groups is

(10) $$\begin{array}{c} F(\pi_{1},\pi_{2})=1. \end{array}$$

Taking into account that $\pi_{1}=C/N$, we can write

(11) $$\begin{array}{c} C=N\Phi (\pi_{2}), \end{array}$$

where Φ is some unspecified function. As we are within the scope of the scaling theory, we assume that Φ is a power function. Thus,

(12) $$\begin{array}{c} C\propto N{\left(\frac{\tau }{t}\right)}^{\alpha }, \end{array}$$

where α is a parameter.

### 2.2. The Number of Homocrony Ho and Its Importance for the Spread of an Epidemic

We introduce the number of homochrony. This is a quantity connected to similarity with respect to time. The number of homochrony is defined as follows:

(13) $$\begin{array}{c} Ho=t/\tau \end{array}$$

We will write this number in analogy to the Fourier number for the case of diffusion

(14) $$\begin{array}{c} Fo=\left(D.t\right)/L^{2}, \end{array}$$

where

- *D*: The coefficient of diffusion.
- *t*: The characteristic time for the transmission of the substance.
- *L*: The characteristic scale of the transmission of the substance.

The number of homochrony (connected to the similarity with respect to time) in analogy with Fo is

(15) $$\begin{array}{c} Ho=t/\tau =(\delta t)/N, \end{array}$$

where we assume that *N*, for the case of COVID-19, is analogous to $L^{2}$ for the case of diffusion. δ is analogous to the diffusion coefficient. Then,

(16) τ=N/δ.

τ is a characteristic time for our system, which characterizes the spread of COVID-19 in a population of *N* individuals with ‘velocity’ of spread δ.

We assume below that δ is a constant. From (12), we can write

(17) $$\begin{array}{c} C=N\varphi \left(Ho\right)=C\left(N,Ho\right)\propto N/Ho^{\alpha } \end{array}$$

Let β=−α. Then,

(18) $$\begin{array}{c} C\propto NHo^{\beta } \end{array}$$

Thus, the number of infected persons is a scaling function of the number of homochrony.

Several remarks on (18) follow.

1. τ reflects the permeability of the system for spread of COVID-19.
2. *t* is the absolute time; Ho=t/τ is the dimensionless relative time.
3. ln(C/N)∝βlnHo. Thus, we have a straight line in the (C/N)−Ho plane with the coefficient β.
4. The experimental data from areas of different sizes will have to collapse on a line in the (C/N)−Ho plane. Indeed, we observe such a collapse below. This will be interpreted as a confirmation of the scaling hypothesis for the spread of COVID-19 in the discussed country.
5. One more note about the homochrony number Ho=t/τ is as follows. τ is a characteristic time which can be calculated on the basis of different models. In the case of the SIR model of epidemic spread
  (19) $$\tau =\frac{1}{\beta \langle k^{2}\rangle /\langle k\rangle -\left(\mu +\beta \right)}.$$
  Here, β is the rate of infection, μ is the recovery rate, *k* is the number of links of an individual from the network and 〈k〉 is the average number of these links [94]. The ratio λ=β/μ characterizes the spread of the infection, and the ratio
  (20) $$M=\frac{\langle k^{2}\rangle }{\langle k\rangle },$$
  is connected to the Molloy-Reed criterion for the existence of a giant component in a network [94].

### 2.3. Self-Similarity of the Process of COVID-19 Spread and Presence of Dynamic Scaling

As we have mentioned above, dynamic scaling (1) is a condition for self-similarity. We note the following.

1. Let us substitute in (1) f→C; x→N; t→Ho; z→α=−β. The result is
  (21) $$C\left(N,t\right)=Ho^{\theta }\phi \left(NHo^{\beta }\right).$$
2. We substitute θ=0 and $\phi \left(NHo^{\beta }\right)\propto NHo^{\beta }$ and we obtain (18).
3. Thus, the spread of COVID-19 is a self-similar phenomenon and exhibits dynamic scaling.

### 2.4. The Scaling Window of a COVID-19 Wave

Next, we have to determine the scaling window of a COVID-19 wave and the time parameter τ. The three phases of the wave can be determined with respect to the tangent of the curve of the wave through the point where the velocity of growth is maximum. The phases are as follows.

1. Initial phase: This is from the beginning to the time point $t_{1}$ where the tangent crosses for the first time the C(t) line.
2. Saturation phase: This is from the time point $t_{2}$ where the tangent crosses the C(t) line for the second time.
3. Scaling phase: This is between the time points corresponding to the two crossings between the tangent and the C(t) line.

We define the characteristic time τ to be

(22) $$\tau =t_{1}+\frac{t_{2}-t_{1}}{e},$$

where *e* is the well-known Euler number. The scaling window connected to the COVID-19 wave will be from $t=t_{1}$ to $t=t_{2}$.

### 2.5. COVID-19 and Allomeric Growth

We will assume that the growth of COVID-19 infection is allometric. Allometric growth means that different parts of a system have different relative rates of growth, but the relative growth rate of each part is constant in a certain period of time [95]. If the system follows the law of allometric growth, the relationship between two parts can be described with a power function, such as

(23) $$y_{t}=ax_{t}^{b}.$$

Here, $x_{t}$ and $y_{t}$ are the measures of the two parts at the time *t*. *a* is the proportionality coefficient and *b* is the scaling exponent. We will be interested in the case when the growth of $x_{t}$ and $y_{t}$ is a logistic one. In this case,

(24) $$\begin{array}{c} x_{t}=\frac{x_{max}}{1+\left(x_{max}/x_{0}-1\right)\exp\left(-k_{x}t\right)};\;\;\;y_{t}=\frac{y_{max}}{1+\left(y_{max}/y_{0}-1\right)\exp\left(-k_{y}t\right)}. \end{array}$$

Here, $x_{max}$ and $y_{max}$ are the maximum values of *x* and *y*. $x_{0}$ and $y_{0}$ are the initial values of *x* and *y*. $k_{x}$ and $k_{y}$ are the original rates of growth of *x* and *y*, respectively. Let us define $X_{t}=x_{t}/\left(x_{max}-x_{t}\right)$ and $Y_{t}=y_{t}/\left(y_{max}-y_{t}\right)$. Then we obtain

(25) $$Y_{t}=\eta X_{t}^{\sigma }.$$

Here, $X_{0}=x_{0}/\left(x_{max}-x_{0}\right)$ and $Y_{0}=y_{0}/\left(y_{max}-y_{0}\right)$. Moreover, $\sigma =(k_{y}/k_{x})$ and $\eta =Y_{0}X_{0}^{-\sigma }$. η is the proportionality coefficient and σ is the allometric scaling exponent.

For small values of *t* (the initial phase of the process of growth) and for large values of $x_{max}$ and $y_{max}$, we can write $x_{t}\approx x_{0}\exp\left(k_{x}t\right)$ and $y_{t}\approx y_{0}\exp\left(k_{y}t\right)$. The growth in this case is approximately exponential. Then the relationship for the allometric growth becomes (23), where $a=y_{0}/\left(x_{0}^{k_{y}/k_{x}}\right)$ and $b=k_{y}/k_{x}$. Sometimes this *b* is written as $b=D_{y}/D_{x}=k_{y}/k_{x}$. $D_{x}$ and $D_{y}$ in the last relationship are the fractal dimensions associated with *x* and *y*. Thus, the last relationship for *b* associates the spatial parameters of the growth $D_{x}$ and $D_{y}$ with the time parameters of the growth $k_{x}$ and $k_{y}$. This indicates that the geographical process based on time and the geographical patterns indicative of space are different sides of the same coin.

## 3. The Spread of COVID-19 in Bulgaria

### 3.1. Four Large COVID-19 Waves in Bulgaria

We present below an overview of the spread of COVID-19 pandemic in Bulgaria.

**Figure 1:**
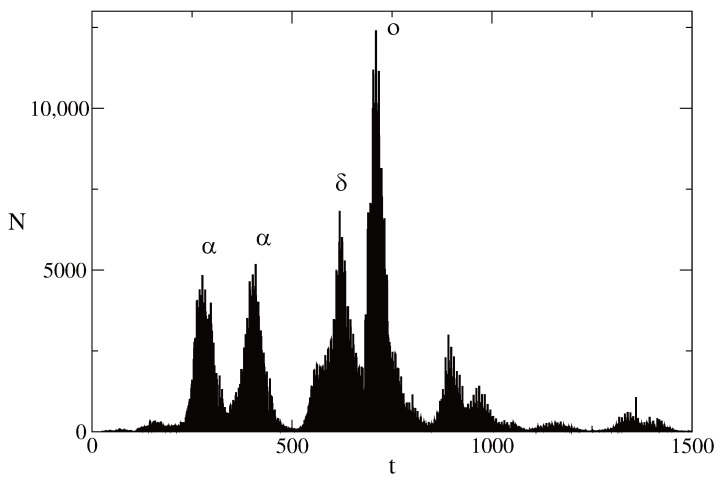
Daily SARS-CoV-2 infections in Bulgaria (15 February 2020–12 April 2024). The four large COVID-19 infection waves due to the α, δ and *o* variants of SARS-CoV-2 are marked by their corresponding symbols. These waves happened at the end of 2020 (the first one), in 2021 (the second and the third one) and at the beginning of 2022 (the fourth one).

Figure 1 shows the daily new cases of COVID-19 infections in Bulgaria in the time interval between 15 February 2020 and 12 April 2024. We note that there was not a large number of infected individuals till the autumn of 2020. The first large peak in the number of infected individuals happened around the end of 2020. The infection was caused by the α version of SARS-CoV-2. The second large wave of infected individuals was observed in the first half of 2021. This wave was also caused by the α version of SARS-CoV-2. The δ version of the virus caused a large wave of infected individuals in the second half of 2021. The largest wave of infected individuals was caused by the *o* version of the virus. This wave occurred at the beginning of 2022. The maximum number of new infections per day for this wave was around 12,000 (the population of Bulgaria was about 6.5 million people at that time). Several smaller waves followed in 2022, 2023 and 2024.

**Figure 2:**
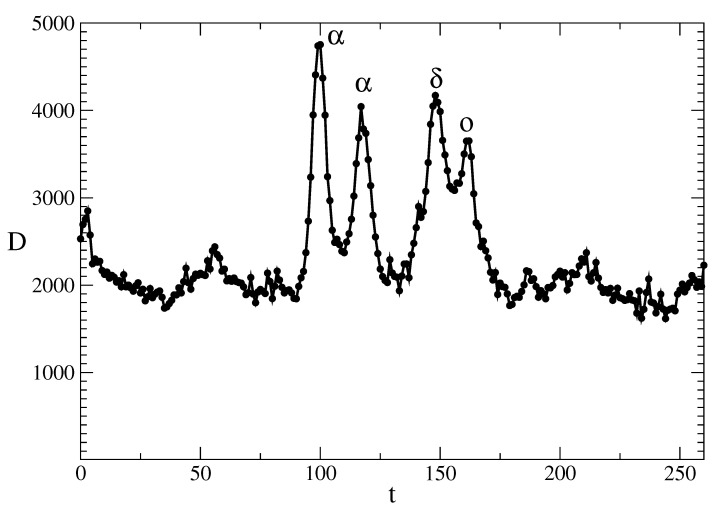
Total weekly deaths in Bulgaria (2019–2023). The four large COVID-19 death waves due to the α, δ and *o* variants of SARS-CoV-2 are marked by their corresponding symbols. These waves happened at the end of 2020 (the first one), in 2021 (the second and the third one) and at the beginning of 2022 (the fourth one).

Figure 2 shows the number of weekly deaths in Bulgaria during the period 2019–2023. We note that the first COVID-19 cases in Bulgaria were registered on the 8th of March 2020 and the situation started to slowly return to normal in the summer of 2022. We also note the following:

1. There was no large wave of COVID-19 infections in the spring of 2020. Because of this, there was a very small spring wave of COVID-19 deaths in 2020.
2. The first large wave of deaths because of COVID-19 occurred at the end of 2020. This was the largest wave of deaths. At that time, no vaccination was available. This wave was caused by the α version of SARS-CoV-2 virus.
3. In 2021, there was another wave of deaths caused by the α version of the SARS-CoV-2 virus. The peak number of deaths for this wave was smaller than the peak number of deaths for the first wave. The reason for this was the better treatment of the infected individuals and the availability of vaccines against the α version of the SARS-CoV-2 virus.
4. The third peak of deaths because of the COVID-19 pandemic was in the second half of 2021 when the dominant variant was the δ version of the SARS-CoV-2 virus.
5. The fourth peak of deaths was at the end of 2021 and at the beginning of 2022, when the *o* version of SARS-CoV-2 virus was dominant in Bulgaria. The number of people infected with COVID-19 in this period was the largest for the entire time interval from 2020 to 2023. The appropriate treatment of the infected individuals and the availability of the vaccination, however, led to a lower peak in the number of deaths in comparison to the first peak from 2020 when the vaccination was still not present.

### 3.2. Analytical Results Obtained from the Data

We study below the first large wave of infections in Bulgaria, which happened at the end of 2020. This wave was not influenced by vaccinations, and the infection spread in the Bulgarian population without many countermeasures. Thus, this wave supplied the best basis for test of our basic hypothesis about the spread of COVID-19 in Bulgaria.

**Figure 3:**
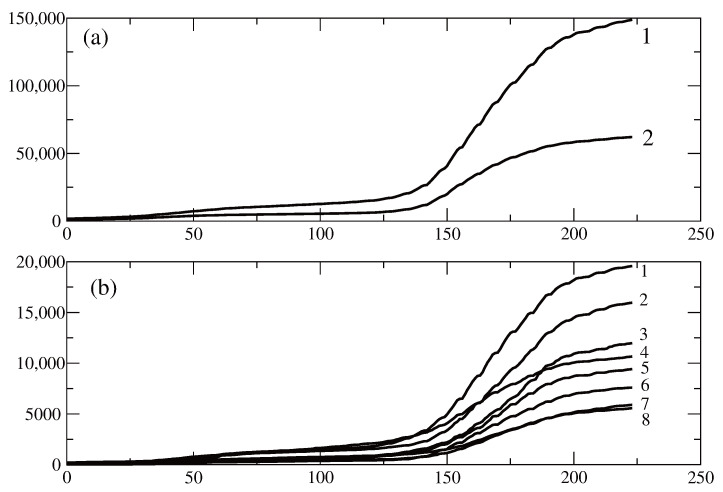
Number of infected individuals as a function of time in Bulgaria and several Bulgarian regions from 6 June 2020 to 15 January 2021. (**a**): Line 1—total number of people infected in Bulgaria; line 2—total number of people infected in the region Sofia-mega which comprises the regions Sofia-city and Sofia-region. (**b**): The graphs are as follows. 1—region of Plovdiv; 2—region of Varna; 3—region of Burgas; 4—region of Blagoevgrad; 5—region of Stara Zagora; 6—region of Ruse; 7—region of Pleven; 8—region of Pazadzhik.

Figure 3 shows data for the period from 6 June 2020 to 15 January 2021. The data is for the entire country and for nine of the largest regions of Bulgaria-Plovdiv, Varna, Burgas, Blagoevgrad, Stara Zagora, Pleven, Pazardzhik and the region Sofia-mega. This last region comprises two regions, Sofia-city and Sofia-region, in order to have all regions be of the same kind—a large central city and an area with smaller towns and villages around the large city. Figure 3a shows the function C(t) for the case of the entire country (solid line) and for the region Sofia-mega (dashed line). We observe here that around day 150 after the beginning of the time period, the number of infected individuals in the country as a whole increases at a higher rate in comparison to the Sofia-mega region. Figure 3b shows the changes in the other eight regions. The final values of C(t) on 15 January 2021 are ranged according to the population *N* of the corresponding regions.

**Figure 4:**
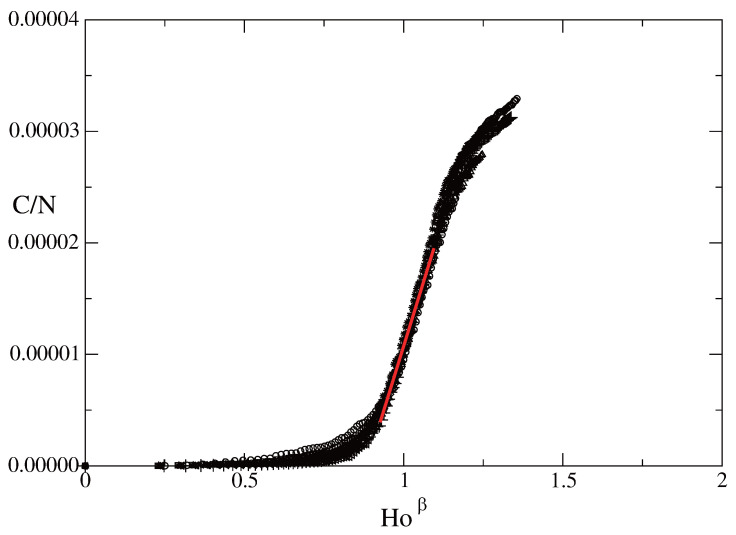
Rescaled functions of the total number of people infected *C* as a function of time: the $\left(C/N\right)-Ho^{\beta }$ functions for several Bulgarian regions for β=2/6. Circles: Region of Blagoebgrad. Squares: Region of Burgas. Diamonds: Region of Pazardzhik. Triangles pointing up: Region of Pleven. Triangles pointing left: Region of Plovdiv. Triangles pointing right: Region of Ruse. Plusses: Region Sofia-mega (Sofia-city and Sofia-region). X: Region Stara Zagora. * —Region Varna. Red line: Average line of collapse in the scaling window of the data. Fitting method for the red line: linear regression. Regression coefficient: 9.239 × $10^{-5}\;\pm \;9.994\;\times \;10^{-7}$. Regression constant: $-8.166\;\times \;10^{-5}\;\pm \;1.010\;\times \;10^{-6}$.

Let us apply the above theory to data from the first COVID-19 wave of infected individuals in Bulgaria in the autumn and winter of 2020–2021. The collapse of the data on the basis of a relationship of the kind $C/N\propto Ho^{\beta }$ is shown in Figure 4.

Figure 4 illustrates the correctness of the hypothesis and the presence of scaling for the scaling window connected to the first wave of COVID-19 in Bulgaria. We observe a clear collapse of the data for all nine regions in the rescaled plane $\left(C/N\right)-Ho^{\beta }$. The data collapses around a curve which is a straight line, and this is another confirmation of the hypothesis of the presence of scaling. We observe a well-formed scaling window.

The following notes are in order with respect to the collapse of the data shown in Figure 4.

1. The form of the relationship for the line of collapse of the data in the scaling window (the red line in Figure 4) can be written as
  (26) $$\frac{C}{N}=k^{2}Ho^{2/6}.$$
2. *k* can be interpreted as a quantity connected to the average number of links of a node in the network of COVID-19 spread in Bulgaria. A closer look at the data shows that for the different regions the regional values of this parameter are $k\propto N^{1/6}$, where *N* is the population of the corresponding region. This result is in agreement with the results concerning the existence of scaling of human interactions with size of population in the case of urban systems [96].

## 4. Concluding Remarks

1. In this text, we formulate a hypothesis about the spread of COVID-19 in Bulgaria. The hypothesis is that the spread of COVID-19 has scaling properties and obeys a power law with respect to the homochrony number.
2. We develop a corresponding theory on the basis of this hypothesis and arrive at the relationship $C\propto NHo^{\beta }$ for the number of people infected in any of the regions of the country. This means that in a coordinate plane with the appropriately selected units for the quantities measured by the axes, we will observe (in the corresponding scaling window) an approximately straight line in all relationships for the number of infected individuals *C* in the regions of the country. Moreover, we observe a collapse of all such straight lines around a single line.
3. We prove the above hypothesis by means of real data. From the four large waves of infection connected to COVID-19, we selected the first COVID-19 wave which was observed in the autumn and in the winter of 2020–2021. This wave was unaffected by the vaccination and other circumstances which could limit the spread of infection. The collapse is indeed observed in the case of real data from the nine largest regions in Bulgaria
4. Finally, we note that the presented results are only first remarks about our studies connected to the scaling theory of COVID-19. In our future research we will study the other waves of COVID-19 infection which are affected by the presence of vaccines. We will also expand our observations to other countries.

## Data Availability

No new data were created or analyzed in this study.
